# Genomic and transcriptomic differences in community acquired methicillin resistant *Staphylococcus aureus* USA300 and USA400 strains

**DOI:** 10.1186/1471-2164-15-1145

**Published:** 2014-12-19

**Authors:** Marcus B Jones, Christopher P Montgomery, Susan Boyle-Vavra, Kenneth Shatzkes, Rosslyn Maybank, Bryan C Frank, Scott N Peterson, Robert S Daum

**Affiliations:** J. Craig Venter Institute, 4120 Capricorn Lane, La Jolla, CA 92037 USA; Department of Pediatrics, Section of Critical Care, University of Chicago, Chicago, IL 60637 USA; Department of Medicine, Center for Emerging and Re-emerging Pathogens, New Jersey Medical School, Rutgers Biomedical and Health Sciences, Rutgers University, Newark, NJ 07103 USA; Battelle National Biodefense Institute, National Biodefense Analysis and Countermeasures Center, Frederick, MD 21702 USA; J. Craig Venter Institute, 9704 Medical Center Drive, Rockville, MD 20850 USA; Sanford Burnham Medical Research Institute, 10901 N. Torrey Pines Rd, La Jolla, CA 92037 USA; Department of Pediatrics, Section of Infectious Diseases, Chicago, IL 60637 USA

## Abstract

**Background:**

*Staphylococcus aureus* is a human pathogen responsible for substantial morbidity and mortality through its ability to cause a number of human infections including bacteremia, pneumonia and soft tissue infections. Of great concern is the emergence and dissemination of methicillin–resistant *Staphylococcus aureus* strains (MRSA) that are resistant to nearly all β-lactams. The emergence of the USA300 MRSA genetic background among community associated *S. aureus* infections (CA-MRSA) in the USA was followed by the disappearance of USA400 CA-MRSA isolates.

**Results:**

To gain a greater understanding of the potential fitness advantages and virulence capacity of *S. aureus* USA300 clones, we performed whole genome sequencing of 15 USA300 and 4 USA400 clinical isolates. A comparison of representative genomes of the USA300 and USA400 pulsotypes indicates a number of differences in mobile genome elements. We examined the *in vitro* gene expression profiles by microarray hybridization and the *in vivo* transcriptomes during lung infection in mice of a USA300 and a USA400 MRSA strain by performing complete genome qRT-PCR analysis. The unique presence and increased expression of 6 exotoxins in USA300 (12- to 600-fold) compared to USA400 may contribute to the increased virulence of USA300 clones. Importantly, we also observed the up-regulation of prophage genes in USA300 (compared with USA400) during mouse lung infection (including genes encoded by both prophages ΦSa2usa and ΦSa3usa), suggesting that these prophages may play an important role *in vivo* by contributing to the elevated virulence characteristic of the USA300 clone.

**Conclusions:**

We observed differences in the genetic content of USA300 and USA400 strains, as well as significant differences of *in vitro* and *in vivo* gene expression of mobile elements in a lung pneumonia model. This is the first study to document the global transcription differences between USA300 and USA400 strains during both *in vitro* and *in vivo* growth.

**Electronic supplementary material:**

The online version of this article (doi:10.1186/1471-2164-15-1145) contains supplementary material, which is available to authorized users.

## Background

Methicillin-resistant *Staphylococcus aureus* (MRSA) isolates have emerged as a leading cause of infectious diseases [1–6]. Once confined primarily to hospitals and patients with defined risk factors, infections caused by community-associated MRSA (CA-MRSA) isolates have become epidemic in the United States and now frequently occur among previously healthy individuals without these risk factors [7, 8]. Although the majority of CA-MRSA infections are mild, some CA-MRSA infectious syndromes such as complicated skin and soft tissue infections, necrotizing pneumonia, bacteremia, and sepsis can be life-threatening [7–10]. After the first report of CA-MRSA deaths in children in it became recognized that the strain type was USA400 [11–14]. During the early emergence of CA-MRSA, isolates of both MRSA and MSSA with the USA400 pulsed field pattern were identified as causes of severe sepsis and death characterized by rapidly progressive clinical deterioration, with necrotizing pneumonia and multiple-organ-system involvement and bilateral adrenal hemorrhage characteristic of the Waterhouse–Friderichsen syndrome [14]. However, among community associated MRSA infections, USA400 has been nearly completely replaced by another CA-MRSA background, called USA300 [15–23]. The reasons for the replacement of USA400 by USA300 are not known.

Many have speculated that the dominance of USA300 is evidence of altered fitness. In support of this notion, USA300 strains are hypervirulent, compared with USA400 strains, in several animal models of skin infection and pneumonia [24]. Although the relationship between fitness and virulence is not clear, understanding the mechanisms of the extraordinary virulence of USA300 strains will shed light onto the reasons for its emergence. The virulent nature of *S. aureus* is mediated by a wide array of cell surface proteins, secreted toxins and mobile genetic elements [15, 25–31]. As such there are at least two possible explanations for the virulence of USA300. One is that USA300 strains have acquired novel genomic content that enhances its fitness and/or virulence. For example, among sequenced *S. aureus* isolates, the arginine catabolic mobile element (ACME) is present in most USA300 strains circulating in the United States, but not USA400 isolates [22]. ACME is a large mobile genetic element also found in some *Staphylococcus epidermidis* isolates encoding at least 33 open reading frames [22, 32]. Deletion of ACME from USA300 enhanced survival in a rabbit model of bacteremia [32], but ACME was not necessary for virulence in rodent models of pneumonia or skin infection [33]. However, ACME encodes an arginine deiminase gene, called *arcA* that allows for enhanced survival in acidic environments [34]. Interestingly, this process drives the synthesis of host polyamines that are toxic to *S. aureus*. It is likely not a coincidence that ACME therefore also encodes a spermine acetyl transferase, *speG*, which counteracts the toxic effects of polyamines. In this regard, ACME appears to at least partially account for the observed pathogenesis of USA300 in skin infection [35, 36]. Nevertheless, ACME-deficient USA300 clinical isolates have been described supporting the data from animal models that ACME is not a necessary component of pathogencity or survival in humans.

An alternative explanation is that USA300 has evolved to alter the expression/activity of *S. aureus* regulatory genes that are part of the core genome. In support of this idea, USA300 isolates have increased expression of the global regulatory systems *agr* and *saeRS,* and attendant increased expression of downstream toxins such as the Panton-Valentine leukocidin (PVL, encoded by lukSF-PV), the alpha-hemolysin (Hla, encoded by *hla*), and phenol soluble modulins (PSMs), compared to USA400 [24, 37, 38]. Although there is a strong epidemiologic association of PVL carriage with severe disease caused by CA-MRSA, its role in pathogenesis is controversial. Since PVL is present in both USA300 and USA400 isolates, it is not likely that its presence accounts for the differences in virulence between the genomic backgrounds. In contrast, *agr*, *saeRS*, *hla* and the PSMs which are core genes present in almost all *S. aureus* strains, clearly advance the pathogenesis of USA300 in animal models [37, 39, 40]. Although it is known that USA300 has altered regulation of selected global regulators and virulence genes compared with USA400, it is not known whether there are global differences in the transcription profiles between USA300 and USA400.

By comparing the *in vitro* transcriptional profiles of USA300 and USA400 using DNA microarray technology, we tested our hypothesis that USA300 strains have global regulatory alterations that contribute to their hypervirulent phenotype. To identify the possibility that the *in vivo* environment in host tissues can influence bacterial gene expression, we quantified bacterial gene expression in the lungs of animals infected with MRSA strains USA300 and USA400. Together our data offer a glimpse into CA-MRSA USA300 and USA400 pathogenesis and suggest a potential model for the observed increased virulence of *S. aureus* USA300 strains.

## Results

### Genomic characterization of USA300 and USA400 strains

To better understand the genomic diversity of USA300 and USA400 strains and the features that distinguish these two lineages, 15 USA300 clinical isolates and 4 USA400 isolates (Table 1) were sequenced using Roche 454 FLX sequencing technology. The completely sequenced USA300_FPR3757 strain was used as a reference for comparison to USA300 isolates, whereas USA400 strains were compared to *S. aureus* USA400 strain MW2. These comparisons allowed us to identify 2,279 genes that are uniformly conserved between all of the sequenced USA300 and USA400 isolates. The number of genes unique to either lineage was similar. USA400 genomes uniquely encode 57 genes (Additional file 1: Table S1) across all isolates, whereas USA300 isolates uniquely encode 46 genes (Additional file 2: Table S2) across all isolates. These genes were examined in greater detail by assessing their predicted functional roles. Novel genes present in USA300 include a toxin-antitoxin system, the chemotaxis-inhibiting protein (CHiPS), and a number of hypothetical proteins in the ΦSa3usa phage. Each of these sequences displays an overall strong conservation within USA300 isolates. Specifically, there were 35 genes that were unique to USA300 isolates compared to USA400 isolates in the ΦSa3usa phage, as well as the ACME element. All USA300 and 400 isolates were also compared to a variety of *S. aureus* sequence-types in a PanGenome analysis (Additional file 3: Tables 3 and Additional file 4: Table S4; Additional file 5: Figure S1). Pangenome analysis revealed 2 major branches (Additional file 5: Figure S1). These branches demonstrate strong clustering of our USA300 and USA400 lineages, compared to other sequence-type (Additional file 3: Table S3). Genes specific to the USA300 lineage appear to be associated with bacteriophage and mobile genomic elements present in USA300 that are absent in the USA400 lineages. Furthermore, kSNP analysis of core genes within our USA300 and 400 lineages (Additional file 6: Table S5; Additional file 7: Figure S2) demonstrate a strong correlation with *S. aureus* strain sequence type.

**Table 1:** **USA300 and USA 400 isolates**

USA Pulsotype	Strain name	ST	coverage	Year	Site of isolation or syndrome	Onset	SRA ID
USA400	staph649	ST1	99.02	2004	Skin infection	CA	SRX026443
USA400	gsast11506	ST1	99	2000		CA	SRX026318
USA400	gsast2674	ST1	99	2000	Respiratory, severe sepsis nec pneumonia	CA	SRX025928
USA400	gsastq2352	ST1	99	2000	Respiratory, severe sepsis nec pneumonia	CA	SRX025931
USA300	gsast10243	ST8	98.15	2007	Blood	CA	SRX026310
USA300	gsast10329	ST8	99.27	2007	Peritoneal/body fluid	HA	SRX026315
USA300	gsast1135	ST8	98.15	2004	Skin infection	CA	SRX026317
USA300	gsast11578	ST8	99.3	2002		CA	SRX025942
USA300	gsast1185	ST8	96.65	2004	Skin infection	CA	SRX025925
USA300	gsast13205	ST8	97.37	2008	Blood, nec pneumonia, sepsis		SRX025943
USA300	gsast2145	ST8	99.28	2005	Skin infection	CA	SRX026311
USA300	gsast5319	ST8	99.32	2005	Skin infection	CA	SRX026314
USA300	gsast9376	ST8	97.91	2007	Respiratory	HA	SRX025927
USA300	gsast9549	ST8	99.26	2007	Screen	HA	SRX025926
USA300	gsast9636	ST8	99.28	2007	Respiratory	HA	SRX025944
USA300	gsast9750	ST8	98.82	2007	Screen	HA	SRX025939
USA300	gsastca11	ST8	95.94	1998		CA	SRX025929
USA300	staph923	ST8	99.31	2004	Skin infection	CA	SRX025930

### *In vitro*differential gene expression between USA300 and USA400 isolates

**Figure 1:**
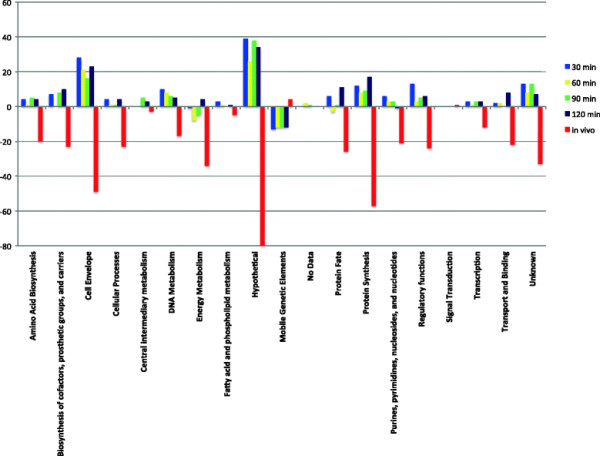
**Role category analysis of** ***S. aureus*** **USA300 and USA400 genes differentially expressed** ***in vitro*** **and** ***in vivo*** **growth.** X- axis represents individual role categories based on gene function. Y-axis values indicate the mathematical difference in the number of genes significantly differentially expressed between USA300 and USA400 isolates during *in vitro* and *in vivo* growth. Positive values indicate a higher number of genes differentially expressed in the corresponding role category in USA300 isolate 923 compared to USA400 isolate 649. Negative values indicate a higher number of genes differentially expressed in the corresponding role category in USA400 isolate 649 compared to USA300 isolate 923. Blue bars indicate values from the 30 min *in vitro* time point; Yellow bars indicate 60 min *in vitro* time point; Green bars indicate 90 min *in vitro* time point; Maroon bars indicate 120 min *in vitro* time point; and Red bars indicate bars indicate the *in vivo* time point.

In order to test our hypothesis that USA300 and USA400 strains differ in their global gene expression patterns, we compared global gene expression between a clinical USA300 isolate (strain 923) and a clinical USA400 isolate (strain 649) using DNA microarrays. We compared gene expression patterns of cells grown in TSB medium at 30, 60, 90, and 120 minutes of growth. An analysis of significant differences in gene expression using SAM revealed 480 genes (342 genes more highly expressed in USA300 isolate and 138 genes more highly-expressed in the USA400 strain. Role category analysis of genes considered to be part of the shared core USA300/USA400 genomes demonstrated an overall higher gene-expression profile in most functional categories in the USA300 isolate compared with the USA400 isolate (Figure 1). The USA300 isolate displayed increased expression for genes encoding cell envelope proteins (including several lipoproteins and superantigen-like proteins) and a large number of hypothetical and genes of unknown function compared with the USA400 isolate 649. Interestingly, genes belonging in mobile elements were more highly expressed in the USA400 strain compared to the USA300 isolate.

Among the genes with differential expression between the strains, there is higher expression in transcription of regulatory genes including *agrBDC* and *rot*, and differential expression of the two prophages present in the genomes of the sequenced USA300 and USA400 genomes. Interestingly, among the genes deemed significantly differentially expressed that reside in the prophage ΦSa2usa (Additional file 8: Table S6), 20 were more highly expressed in the USA400 isolate, whereas 6 genes were more highly expressed in the USA300 strain. In contrast, the genes residing in prophage ΦSa3usa were highly expressed in the USA300 strain and not detected in the USA400 strain (Additional file 9: Table S7). This is consistent with the fact that the prophage ΦSa3usa insertion was only found to be present in the USA300 isolate and absent in the USA400 isolate, which is consistent with the reference USA300 and USA400 genomes.

Analysis of genes known to play a role in neutrophil evasion functions displayed differential expression (Additional file 10: Table S8). Genes that had significantly higher expression in the USA300 isolate compared with the USA400 strain included: immunoglobulin G binding protein A precursor (SAUSA300_0113), fibrinogen-binding protein (SAUSA300_1055), superantigen-like protein (SAUSA300_0407) and a leukocidin family protein (SAUSA300_1975). Several genes encoded in the pathogenicity islands vSAα and vSAβ also more highly expressed in the USA300 isolate Additional file 11: Table S9). Interestingly, compared with USA400, we observed an overall reduced expression of genes located in the *S. aureus* pathogenicity island SaPI5, with the exception of the gene encoding the *ear* exoprotein that is associated with virulence in USA300. There were also a number of lipoproteins and proteases more highly expressed in the USA300 isolate compared with USA400 strain. The gene encoding the IgG binding protein, Sbi, displayed slightly increased expression in the USA300 isolate. Surprisingly, expression of *agrA* and the δ-hemolysin were more highly expressed in the USA400 strain. Other genes of interest that were more highly expressed in the USA400 strain included two antibacterial proteins and *lukF-PV*.

### *In vivo*differential gene expression comparison of USA300 and USA400

Because it is not known if *in vitro* gene expression correlates with that seen *in vivo,* we sought to determine the differential expression of USA300 and USA400 isolates *in vivo* in a mouse model of lung infection by using complete genome qRT-PCR analysis. We chose to assess *in vivo* expression of bacteria during infection of lungs of mice 4 hours after inoculation with *S. aureus* because the number of bacteria recovered from the lungs at this time point is the same, regardless of the infecting isolate, because all animals are sick at this time, and because it is before the onset of mortality. Consistent with this, all infected animals appeared ill at 6 hours post-infection. Our results recapitulate other virulence studies as, nearly all (4/5) mice infected with USA300 strains died within 24 to 48 hours after inoculation, whereas none (0/5) of the mice infected with USA400 strains died. When using a cutoff of two-fold change in expression, we observed 624 genes with higher expression in the USA300 isolate compared to 1075 genes displaying elevated expression in the USA400 strain. Among these USA300 genes were encoded in ACME, the prophages ΦSA2usa and ΦSA3usa, the pathogenicity islands vSAα and vSAβ, and genes *arcC*, *argR*, and *coa*. These data suggest that, although the USA300 isolate has increased *in vitro* expression compared with USA400 strains, the converse is true *in vivo* (Figure 1).

As we observed in the *in vitro* expression analysis, there were striking differences in the expression of the prophages ΦSA2usa and ΦSA3usa between USA300 and USA400 *in vivo* (Additional file 8: Table S6 and Additional file 9: Table S7). In concordance with the *in vitro* data, genes in ΦSA3usa were more highly expressed during lung infection in the USA300 isolate compared with the USA400 strain. However, whereas genes encoded in ΦSA2usa were generally expressed at higher levels in USA400 compared with USA300 *in vitro*, the *in vivo* expression was generally higher in the USA300 isolate. This suggests that *in vivo* cue(s), and a potentially novel signal transduction network, is required for full expression of ΦSa2usa, and other virulence genes, in *S. aureus* USA300.

The *in vivo* expression of genes contained in ACME was detected in lungs infected with USA300 but not USA400. This was expected and validates our DNA sequence data showing that ACME is not present in the USA400 strain. As we observed *in vitro*, there were a number of lipoproteins whose expression during lung infection was higher in USA300 compared with USA400. Interestingly, there was strongly induced expression of the epidermin immunity protein operon after infection with USA300, compared with USA400. This operon has been previously associated with increased fitness of CA-MRSA strains due to its role in lantibiotic resistance [41]. Notably, there was also higher expression of a cluster of urease genes in USA300 during infection of lungs compared with USA400. These genes have been demonstrated to be induced during acid shock, and may serve as a mechanism to neutralize acidification of the phagolysosomal compartment and promote intracellular survival [42]. Among the genes in USA400 whose expression increased during lung infection compared with USA300, we were surprised to find the two-component global regulatory system *saeRS* and δ-hemolysin, in part, which is under the control of the *agr* effector, RNAIII. There was also increased *in vivo* expression of the toxin encoding genes *hla*, *hlb*, and *hlc* in USA400 strain (Additional file 10: Table S8).

## Discussion

USA300 MRSA isolates have emerged as the dominant cause of CA-MRSA infections in the United States. However, it has been unclear why USA300 has replaced its predecessor (USA400) in CA-MRSA infection. Furthermore, understanding the rapid evolution that led to increased virulence of *S. aureus* USA300 isolates, compared with highly virulent USA400 isolates, can inform better strategies for treating and preventing CA-MRSA infections. Previous work has suggested that the increased virulence of USA300 isolates might be due to increased expression of global regulators and toxin genes [24]. Although this would explain the hypervirulent phenotype of USA300 isolates, the relationship between virulence and fitness is less well understood. In an effort to understand the contribution of genetic factors that contribute to the success of USA300, we performed comparative genome sequencing of USA300 and USA400 isolates, *in vitro* gene expression analysis, and assessed *in vivo* gene expression by means of complete genome qPCR in an established mouse model of *S. aureus* necrotizing pneumonia.

Analysis of the sequenced isolates revealed a strong conservation of genes between ST1 (USA400) and ST8 (USA300) isolates as expected. However, genes that were deemed unique to USA300 isolates include genes encoded in the ACME element, the prophage ΦSA3usa and a toxin-antitoxin system. However, many of these genes are not functionally characterized and are annotated as hypothetical with no defined function. It is well documented that mobile elements often encode genes that provide a fitness advantage in the form of antibiotic resistance determinants, toxins and intracellular survival determinants. It is plausible that many of the hypothetical genes we identified that are unique to USA300 isolates may have provided an *in vivo* fitness advantage and increased the virulence compared with USA400 and other *S. aureus* isolates.

Although *S. aureus* gene expression was globally higher in isolate USA300 compared with USA400 *in vitro*, expression was globally higher *in vivo* during lung infection in USA400 isolate 649 compared with USA300 isolate 923. In some cases, there was higher expression of lipoproteins in USA300 compared with USA400 during infection, just as there was *in vitro*. Expression of the ACME element was also increased in USA300 after infection, compared with USA400; this was expected because ACME is only present in USA300 but it serves as a validation of the experiment. There was also increased expression of *agrB*, *agrD*, and ΦSA3usa in USA300 during infection of lungs compared with USA400, in concordance with the *in vitro* data. Also to note was the increased expression of *agrA* and *hla* in USA400 compared with USA300 during *in vitro* growth. Previously, we observed increased *in vitro* expression of both *agrA* and *hla* in USA300 compared with USA400, however that observation was at a later time point during infection [39].

On the other hand, expression of *saeRS* and δ-hemolysin (encoded within *agr*) was higher in USA400 during infection compared with USA300. Both *sae* and *agr* are necessary for virulence in *S. aureus* necrotizing pneumonia caused by USA300 and it is likely that these are also important in USA400 necrotizing pneumonia [43]. There was also increased expression of the *agr-* and *saeRS-* controlled toxin genes *hla*, *lukS-PV*, and 2 PSMs in USA400 compared with USA300 during infection. Although USA400 has a higher LD_50_ than USA300 in rodent experimental models, it is nevertheless capable of producing a high mortality rate in groups of mice infected at its lethal dose [24]. Moreover, when USA400 was still in circulation among humans in the United States, it was highly virulent as it was recognized for causing severe sepsis and rapidly progressive disease leading to death [14]. Therefore, The finding that *agr-* and *saeRS-* controlled toxin genes *hla*, *lukS-PV*, and 2 PSMs were expressed at higher levels in USA400 in this study reinforces the observation that USA400 was a virulent strain of *S. aureus* in humans. We note that our study is a single time point, and *S. aureus* gene expression may differ significantly at different stages of growth/infection [44].

One possible scenario for the discordance in *S. aureus* gene expression during *in vitro* and *in vivo* growth is the absence of complex host/immune factors that act as environmental cues resulting in modulation of *S. aureus* gene expression. Data generated from *in vitro* growth conditions have provided the wealth of knowledge that has given the scientific community the foundation required to understand the complex regulatory networks that modulate signaling/expression pathways. However, conclusions drawn from *in vitro* gene expression studies must be interpreted with caution as numerous details of the *S. aureus* transcriptome may differ significantly between *in vitro* and *in vivo* growth as our study attests. Thus, effort must be made to measure virulence gene expression of signal transduction networks directly from infection models using approaches similar to the one described in this study if we are to identify vaccine targets relevant to infection.

An intriguing possibility of the features bestowed to USA300 strains to be successful may be related to increased emphasis on immune evasion during infection. This hypothesis is supported by the increased expression of ΦSA3usa observed after infection with USA300 compared with USA400. ΦSA3usa encodes a number of immune evasion genes, including CHIPs and staphylokinase [22]. There was also higher expression of the epidermin immunity protein operon in USA300 during infection, compared with USA400. This cluster of genes has been previously associated with increased fitness of CA-MRSA strains due to its role in lantibiotic resistance. Along these lines, there was higher expression of genes encoding urease subunits and metabolism in USA300 during infection compared with USA400. These genes have been demonstrated to be induced during acid shock, and may serve as a mechanism to neutralize acidification of the phagolysosome and promote intracellular survival and fitness. Therefore, USA300 may exploit increased urease activity under low pH, to neutralize the phagolysosomal compartment, allowing USA300 isolates to survive an acute immune response and may explain why USA300 is associated with persistent and recurrent infections. Our hypothesis is supported by recent observation of a *S. aureus* strain that inhibits acidification of a phagolysosome following phagocytosis [45].

## Conclusion

Our findings have important implications in the characterization of pathogenesis and virulence effectors in *S. aureus*. The discordant findings between the *in vitro* and *in vivo* settings suggest that studies to characterize *S. aureus* virulence should attempt to mimic the hostile host environment (e.g. pH shock); and must be comparatively analyzed with *ex vivo* and *in vivo* time-course models to fully reconstruct the arsenal of virulence factors and pathogenesis of *S. aureus*. Complete understanding of the host-pathogen signal interaction during acute and persistent infections is critical for the treatment and prevention of CA-MRSA and all other *S. aureus* infections. In summary, there are differences in the genetic content of USA300 and USA400 strains. This is the first study to document the global transcription differences between USA300 and USA400 strains during both *in vitro* and *in vivo* growth.

## Methods

### Bacterial strains

Strain 649 is a well characterized USA400 MRSA isolate (SCC*mec* type IV, *pvl*+) that belongs to multilocus sequence type (ST) 1. This isolate was obtained from the respiratory tract of an infant with necrotizing pneumonia who died of severe sepsis [12, 14]. Strain 923 is a USA300 MRSA isolate (SCC*mec* type IV+, *pvl*+) that belongs to ST8 [46] and carries the arginine catabolic mobile element (ACME) [33]; it was obtained from a skin abscess. Strain 923 is more virulent than strain 649 in lung and skin infection rodent models [24]. Additional strains sequenced are shown in Table 1.

### Genome sequencing and assembly

All strains were grown in tryptic soy broth (TSB) and genomic DNAs were isolated. All genomic DNAs were sequenced using the Roche 454 sequencing platform. The sequences of strains USA300 and USA400 were assembled using Newbler from the Roche 454 software suite. Quality assessment of the assembled genomes was based on depth of coverage calculated from the ACE files and also breadth of coverage by mapping to their respective reference genomes. All strains were annotated using the Manatee tool developed at JCVI. All reads were submitted to SRA (Table 1.)

### Bacterial growth and RNA isolation from *in vitro*growth

*S. aureus* RNA was isolated from *in vitro* bacterial cultures grown in TSB at 30, 60, 90 and 120 minutes post inoculation. Two volumes of RNA protect (Qiagen) was directly added to the growth media and subsequently pelleted and stored at −20°C until RNA extraction, using the Ambion mirVana RNA kit (Austin, TX). RNA quantity and quality was assessed by measuring total RNA on a nanodrop and visualizing RNA on an agarose gel. Purified RNA was stored at −80°C. Murine lungs harvested from infected mice were homogenized in the Ambion Mirvana lysis-binding buffer and further extracted using the PCT pressure cycler (Pressure Biosciences). Total RNA was extracted from homogenized and lysed tissues as described by the manufacturer (Ambion).

### Generation of probes for microarray experiments, microarray hybridization, normalization and data analysis

DNA probes for microarray experiments were generated by adding 2 μg of total RNA to a mixture containing 6 μg of random hexamers (Invitrogen), 0.01 M dithiothreitol, an aminoallyl-deoxynucleoside triphosphate mixture containing 25 mM each dATP, dCTP, and dGTP, 15 mM dTTP, and 10 mM amino-allyl-dUTP (aa-dUTP) (Sigma), reaction buffer, and 400 units of SuperScript III reverse transcriptase (Invitrogen) at 42°C overnight. The RNA template was then hydrolyzed by adding NaOH and EDTA to a final concentration of 0.2 and 0.1 M, respectively, followed by incubatation at 70°C for 15 min. Unincorporated aa-dUTP was removed with a QIAquick column (Qiagen). The probe was eluted with PE buffer (4 mM KPO_4_, pH 8.5, in ultrapure water), dried to completion, and resuspended in 0.1 M sodium carbonate buffer (pH 9.0). To couple the amino-allyl cDNA with fluorescent labels, Cy3 or Cy5 mono-reactive dye (Amersham) was added for 1 h. Uncoupled label was removed using the Qiagen QIAquick PCR purification kit (Valencia, CA).

Aminosilane-coated slides representing a set of 4,589 *S. aureus* unique open reading frame sequences derived from seven reference genomes and plasmid, pLW043 (http://www.jcvi.org) were prehybridized in 5× SSC (1× SSC is 0.15 M NaCl plus 0.015 M sodium citrate) (Invitrogen), 0.1% sodium dodecyl sulfate, and 1% bovine serum albumin at 42°C for 60 min. The slides were then washed at room temperature with distilled water, dipped in isopropanol, and allowed to dry. Equal volumes of the appropriate Cy3- and Cy5-labeled probes were combined, dried and resuspended in a solution of 40% formamide, 5× SSC, and 0.1% sodium dodecyl sulfate. Resuspended probes were heated to 95°C prior to hybridization. The probe mixture was then added to the microarray slide and allowed to hybridize overnight at 42°C. Hybridized slides were washed sequentially in solutions of 1x SSC-0.2% SDS, 0.1× SSC-0.2% SDS, and 0.1× SSC at room temperature, then dried in air, and scanned with an Axon GenePix 4000 scanner. Agilent hybridizations were performed as described by the manufacturer. Individual TIFF images from each channel were analyzed with TIGR Spotfinder (available at http://sourceforge.net/projects/mev-tm4/files/mev-tm4/). Microarray data were normalized by LOWESS in TIGR MIDAS software (available at http://sourceforge.net/projects/mev-tm4/files/mev-tm4/). All gene expression data is publicly available at GEO under GSE26255.

### Mouse infection model

All animal experiments were approved by the Institutional Committee on the Care and Use of Animals at the University of Chicago. Our mouse model of *S. aureus* necrotizing pneumonia has been described [39]. Briefly, bacteria were subcultured from frozen stocks onto tryptic soy agar (TSA) and incubated overnight at 37°C. The following evening, one colony was subcultured in 5 ml TSB and incubated overnight with shaking (250 rpm) at 37°C. The following morning, the overnight culture was diluted 1:100 in fresh TSB (flask:volume ratio 7:1) and grown to mid-exponential phase. The bacteria were pelleted by centrifugation (4000 × g, 15 min), washed in sterile PBS, and resuspended in PBS to a final concentration of 2 × 10^8^ CFU/20 μl. The inoculum was confirmed by plating serial dilutions on TSA. Six week old C57Bl/6 mice (10 mice with each isolate) (Jackson labs) were sedated with ketamine and xylazine, after which they were inoculated with 20 μl of the bacterial preparation. Four hours after inoculation, 5 mice from each group were euthanized by forced CO_2_ inhalation. The lungs were removed aseptically and placed in RNAprotect.

### cDNA generation and qPCR assays

qPCR plates were prepared using 1:10 dilutions of a combined stock of forward and reverse primers at a final concentration of 1.25 μM (Invitrogen). 10 μl of diluted 0.125 μM primers was dispensed in quadruplicate to 384-well plates and were subsequently dried. cDNA samples were generated using the overnight method described above to generate probes for microarray experiments without the use of aa-dUTP. 10 ng of each sample was then combined with 2.5 μl of 2X SYBR Green Master Mix (Roche) to a total reaction volume of 5 μl per well. Median Crossing point (Cp) values for the qPCR quadruplicate reactions were calculated. The relative expression changes for each gene were calculated by ΔΔCp normalization based on 4 control genes (SAUSA300_0175, SAUSA300_2543, SAUSA300_2001 and SAUSA300_1747). To determine fold change, the normalized Cp values of USA300 and USA400 gene expression were used to calculate the ΔΔCp during *in vivo* growth. The difference of the gene expression was regarded as significant when ΔΔCp ≥1 or ≤ −1 (>2-fold).

### Availability of supporting data

All microarray gene expression data is publicly available at GEO at the NCBI (GenBank) under GSE26255. All 454 sequencing data may be found in the short read achieve (SRA: http://www.ncbi.nlm.nih.gov/sra/).

## Electronic supplementary material

Additional file 1: Table S1: Gene unique to USA300 lineage compared to USA400 isolates by PanGenome analysis by PanOct. (XLS 75 KB)

Additional file 2: Table S2: Gene unique to USA400 lineage compared to USA300 isolates by PanGenome analysis by PanOct. (XLS 35 KB)

Additional file 3: Table S3: PanGenome analysis comparing USA300 and USA400 isolates to other *S. aureus* sequence types. A “1” designation indicates presence of a gene and a “0” indicates absence. (TXT 3 MB)

Additional file 4: Table S4: Table of genes, based on a p values ≤0.001, that support the 2 major branches. Analysis of these genes reveals a significant number of conserved hypothetical genes. These genes appear to be associated with bacteriophage and mobile genomic elements present in USA300 that are absent in the USA400 lineages. (TXT 36 KB)

Additional file 5: Table S5: Table of SNPs identified by kSNP analysis resulting is clustering of *S. aureus* isolates by sequence type. (PDF 238 KB)

Additional file 6: Figure S1: PanGenome Analysis of USA300 and USA400 lineages compared to other *S. aureus* Sequence-types. A pan-genome analysis of Staphylococcus aureus genomes was done using PanOCT v 2.1 to generate clusters of orthologous genes. This was a broad representation of *Staphylococcus aureus* genomes that included several representatives of USA300 and USA400 sequence types. Green icons indicate USA300 isolates and red icons indicate USA400 isolates. (ZIP 1 MB)

Additional file 7: Figure S2: kSNP analysis of USA300 and USA400 isolates. Single Nucleotide Polymorphism (SNP) analysis was done and phylogenetic trees were created using the software package kSNP v2.1.2. *Staphylococcus aureus* subsp. aureus USA300_FPR3757 (CP000255.1) was the USA300 reference and *Staphylococcus aureus* subsp. aureus MW2 (NC_003923.1) was the USA400 reference. Red coloring represent USA300 isolates and green shading represent USA400 isolates. (PDF 2 KB)

Additional file 8: Table S6: Genes encoded in prophage ΦSa2usa annotated in the USA300 genome were profiled for differential expression compared to USA400 isolate 649 during in vitro and *in vivo* growth. Negative values (green shading) represent genes higher expressed in the USA400 isolate compared to the USA300. Positive values (red shading) represent genes higher expressed in the USA300 isolate compared to the USA400 isolate. #N/A indicates no expression ratio was obtained. Vales are log_2_. (DOCX 133 KB)

Additional file 9: Table S7: Genes encoded in prophage ΦSa3usa annotated in the USA300 genome were profiled for differential expression compared to USA400 isolate 649 during *in vitro* and *in vivo* growth. Negative values (green shading) represent genes higher expressed in the USA400 isolate compared to the USA300. Positive values (red shading) represent genes higher expressed in the USA300 isolate compared to the USA400 isolate. #N/A indicates no expression ratio was obtained. Vales are log_2_. (DOC 127 KB)

Additional file 10: Table S8: Genes involved in neutrophil evasion as annotated in the USA300 genome, were profiled for differential expression compared to USA400 isolate 649 during *in vitro* and *in vivo* growth. Negative values (green shading) represent genes higher expressed in the USA400 isolate compared to the USA300. Positive values (red shading) represent genes higher expressed in the USA300 isolate compared to the USA400 isolate. #N/A indicates no expression ratio was obtained. Vales are log_2_. (DOC 116 KB)

Additional file 11: Table S9: Genes encoded in pathogenicity islands vSAα and vSAβ, as annotated in the USA300 genome, were profiled for differential expression compared to USA400 isolate 649 during *in vitro* and *in vivo* growth. Negative values (green shading) represent genes higher expressed in the USA400 isolate compared to the USA300. Positive values (red shading) represent genes higher expressed in the USA300 isolate compared to the USA400 isolate. #N/A indicates no expression ratio was obtained. Vales are log_2_. (DOC 124 KB)
