# Supplementary figures and images for: Genomic and transcriptomic differences in community acquired methicillin resistant Staphylococcus aureus USA300 and USA400 strains
Source: BMC Genomics. 2014 Dec 19;15:1145. doi: 10.1186/1471-2164-15-1145 (PMC4630920; doi:10.1186/1471-2164-15-1145)

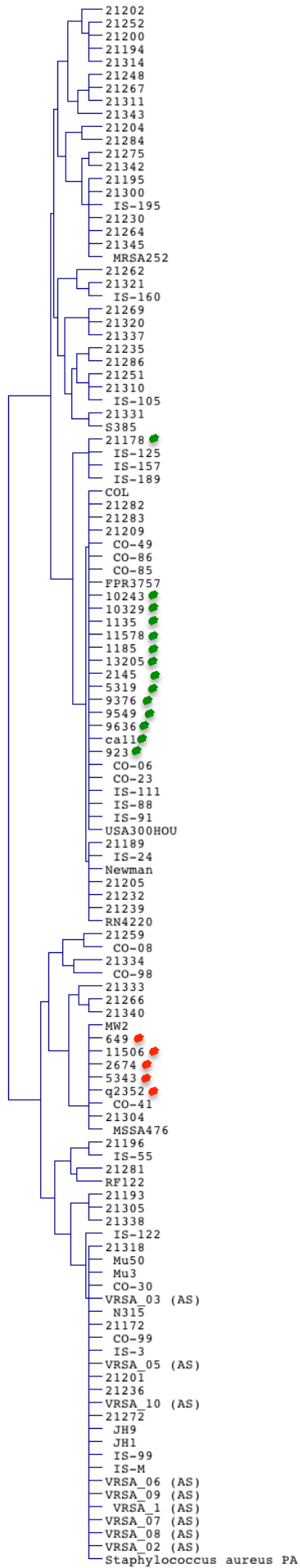

Supplement: Supplementary file 5 — Additional file 5: Table S5: Table of SNPs identified by kSNP analysis resulting is clustering of S. aureus isolates by sequence type. (PDF 238 KB) [file 12864_2014_7077_MOESM5_ESM.pdf]

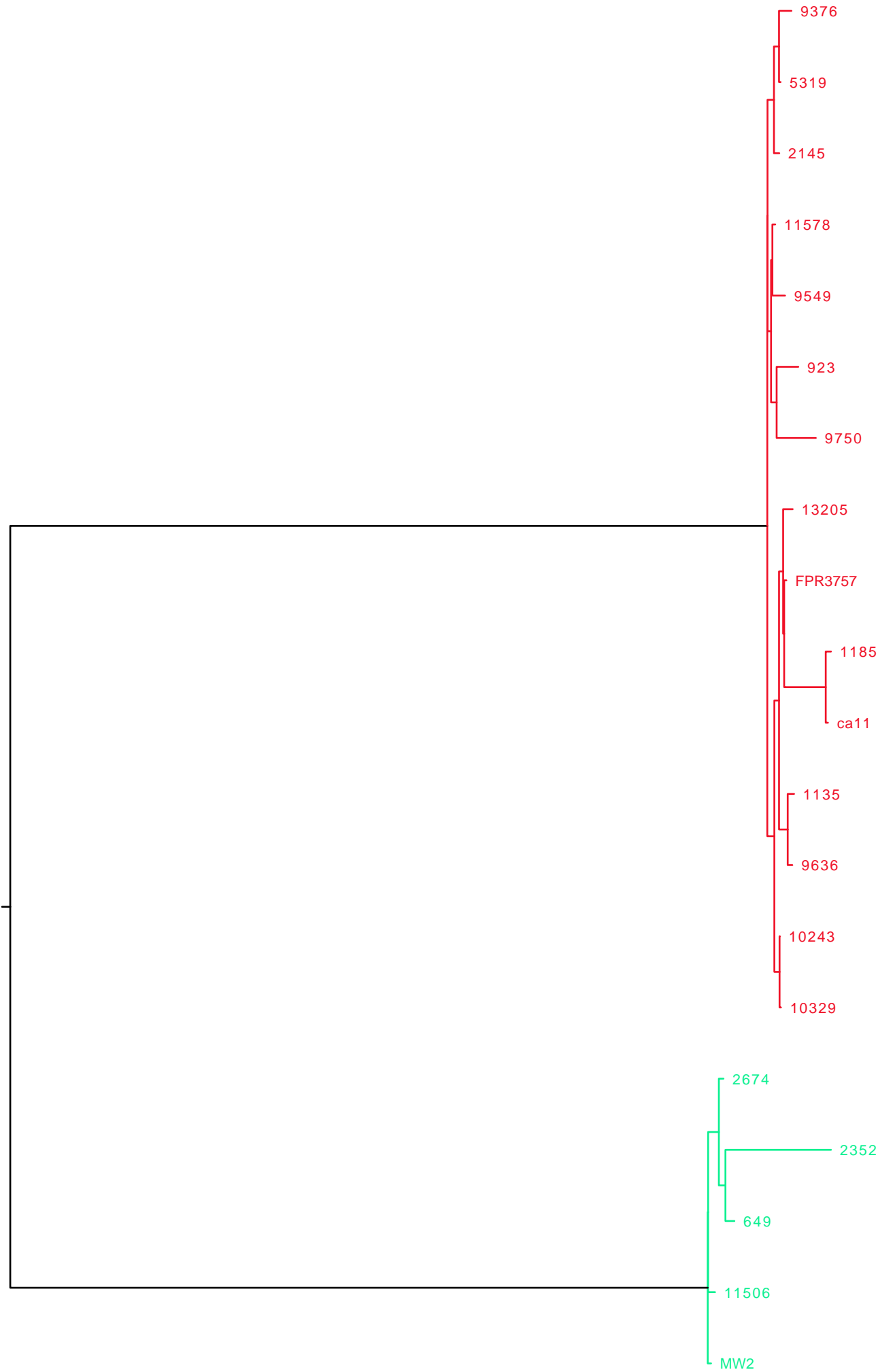

Supplement: Supplementary file 7 — Additional file 7: Figure S2: kSNP analysis of USA300 and USA400 isolates. Single Nucleotide Polymorphism (SNP) analysis was done and phylogenetic trees were created using the software package kSNP v2.1.2. Staphylococcus aureus subsp. aureus USA300_FPR3757 (CP000255.1) was the USA300 reference and Staphylococcus aureus subsp. aureus MW2 (NC_003923.1) was the USA400 reference. Red coloring represent USA300 isolates and green shading represent USA400 isolates. (PDF 2 KB) [file 12864_2014_7077_MOESM7_ESM.pdf]
