# Supplementary material for: Genomic and transcriptomic differences in community acquired methicillin resistant Staphylococcus aureus USA300 and USA400 strains
Source: BMC Genomics. 2014 Dec 19;15:1145. doi: 10.1186/1471-2164-15-1145 (PMC4630920; doi:10.1186/1471-2164-15-1145)
Supplement: Supplementary file 6 [file 12864_2014_7077_MOESM10_ESM.doc]

**Table 4. Genes involved in neutrophil evasion**

| Locus | COMMON_NAME | SYMBOL | 30 | 60 | 90 | 120 | in vivo |
|---|---|---|---|---|---|---|---|
| SAUSA300_0108 | myosin-cross-reactive antigen | | -3.41 | -3.68 | -3.37 | -3.99 | 6.92 |
| SAUSA300_0113 | immunoglobulin G binding protein A precursor | | 0.55 | 0.99 | 1.13 | 2.24 | 2.13 |
| SAUSA300_0380 | alkyl hydroperoxide reductase subunit C | ahpC | 0.78 | 0.27 | 0.43 | 0.11 | -9.14 |
| SAUSA300_0398 | superantigen-like protein | | -0.40 | 0.05 | -0.18 | 0.23 | 0.66 |
| SAUSA300_0407 | superantigen-like protein | | 4.59 | 5.55 | 5.46 | 2.71 | 2.42 |
| SAUSA300_0883 | putative surface protein | | 0.58 | 0.28 | 0.41 | 0.31 | -1.21 |
| SAUSA300_1052 | fibrinogen-binding protein | | -1.53 | -1.83 | -1.53 | -1.76 | -2.55 |
| SAUSA300_1055 | fibrinogen-binding protein | efb | 2.95 | 3.36 | 3.57 | 2.60 | -10.19 |
| SAUSA300_1058 | alpha-hemolysin precursor | | -2.50 | -3.35 | -3.40 | -1.62 | -14.28 |
| SAUSA300_1067 | anti protein | | -2.48 | -3.75 | -2.91 | -2.06 | -17.96 |
| SAUSA300_1255 | oxacillin resistance-related FmtC protein | fmtC | -0.14 | 0.70 | 0.11 | 1.36 | -8.31 |
| SAUSA300_1381 | Panton-Valentine leukocidin, LukF-PV | lukF-PV | -1.02 | -1.01 | -0.55 | -0.96 | -1.41 |
| SAUSA300_1382 | Panton-Valentine leukocidin, LukS-PV | lukS-PV | 0.33 | -0.34 | 0.08 | 0.02 | -2.71 |
| SAUSA300_1445 | hypothetical protein | scpA | 1.35 | 2.28 | 2.50 | 2.24 | 0.97 |
| SAUSA300_1768 | leukotoxin LukD | lukD | -0.11 | -0.06 | 0.63 | -0.01 | -1.71 |
| SAUSA300_1769 | leukotoxin LukE | lukE | #N/A | #N/A | #N/A | #N/A | 1.19 |
| SAUSA300_1890 | staphopain A | | #N/A | #N/A | #N/A | #N/A | -1.95 |
| SAUSA300_1919 | hypothetical protein | | 0.25 | -0.14 | -1.17 | -1.74 | -3.84 |
| SAUSA300_1920 | chemotaxis-inhibiting protein CHIPS | chs | #N/A | #N/A | #N/A | #N/A | 13.11 |
| SAUSA300_1922 | staphylokinase precursor | sak | -0.46 | 0.01 | -0.29 | 0.55 | 2.64 |
| SAUSA300_1974 | leukocidin/hemolysin toxin family protein | | -3.24 | -4.22 | -3.94 | -4.80 | -1.01 |
| SAUSA300_1975 | Aerolysin/leukocidin family protein | | 1.43 | 1.82 | 3.21 | 3.32 | -1.11 |
| SAUSA300_2364 | IgG-binding protein SBI | sbi | 0.95 | 0.90 | 0.55 | 0.44 | -3.25 |
| SAUSA300_2365 | gamma-hemolysin component A | hlgA | -0.41 | -0.33 | -0.23 | 0.45 | -6.06 |
| SAUSA300_2366 | gamma-hemolysin component C | hlgC | #N/A | #N/A | #N/A | #N/A | -2.93 |
| SAUSA300_2367 | gamma-hemolysin component B | hlgB | #N/A | #N/A | #N/A | #N/A | -4.03 |
| SAUSA300_2504 | acyltransferase | | 0.14 | -0.60 | 0.38 | -0.16 | -0.61 |
| SAUSA300_2572 | zinc metalloproteinase aureolysin | aur | 0.52 | 0.55 | 0.56 | 1.01 | 0.82 |

*Negative values (green shading) represent genes higher expressed in USA400 isolate 649 compared to USA300 isolate 923. Positive values (red shading) represent genes higher expressed in USA300 isolate 923 compared to USA400 Isolate 649. #N/A indicate no expression ratio was obtained between isolates. Values are log2 ratios of differential gene expression.
