# Supplementary material for: Genomic and transcriptomic differences in community acquired methicillin resistant Staphylococcus aureus USA300 and USA400 strains
Source: BMC Genomics. 2014 Dec 19;15:1145. doi: 10.1186/1471-2164-15-1145 (PMC4630920; doi:10.1186/1471-2164-15-1145)
Supplement: Supplementary file 7 [file 12864_2014_7077_MOESM11_ESM.doc]

## Table 5. Genes encoded in vSAα and vSAβ

| USA300 | COMMON_NAME | 30 | 60 | 90 | 120 | in vivo |
|---|---|---|---|---|---|---|
| SAUSA300_0391 | hypothetical protein | #N/A | #N/A | #N/A | #N/A | 2.11 |
| SAUSA300_0392 | hypothetical protein | 1.09 | 0.81 | 0.60 | -0.01 | -5.13 |
| SAUSA300_0393 | hypothetical protein | -0.01 | -0.07 | 0.88 | 0.66 | 0.66 |
| SAUSA300_0395 | superantigen-like protein | 2.28 | 2.25 | 1.76 | 1.54 | -1.33 |
| SAUSA300_0396 | superantigen-like protein | #N/A | #N/A | #N/A | #N/A | -0.81 |
| SAUSA300_0397 | superantigen-like protein | 0.07 | 0.69 | -0.07 | -0.32 | 8.00 |
| SAUSA300_0398 | superantigen-like protein | -0.40 | 0.05 | -0.18 | 0.23 | 0.66 |
| SAUSA300_0403 | superantigen-like protein | #N/A | #N/A | #N/A | #N/A | 3.46 |
| SAUSA300_0406 | putative restriction/modification system specificity protein | 2.27 | 2.57 | 2.73 | 2.01 | 4.31 |
| SAUSA300_0407 | superantigen-like protein | 4.59 | 5.55 | 5.46 | 2.71 | 2.42 |
| SAUSA300_0408 | hypothetical protein | 3.47 | 4.06 | 4.42 | 1.57 | #N/A |
| SAUSA300_0409 | hypothetical protein | 1.27 | 0.63 | 0.67 | 0.89 | -9.13 |
| SAUSA300_0415 | staphylococcal tandem lipoprotein | 1.37 | 2.18 | 0.60 | 3.42 | 0.72 |
| SAUSA300_0416 | staphylococcal tandem lipoprotein | 3.06 | 2.18 | 2.25 | 2.79 | -0.15 |
| SAUSA300_0417 | staphylococcal tandem lipoprotein | -0.06 | 0.92 | 0.90 | 0.89 | 2.21 |
| SAUSA300_0418 | staphylococcal tandem lipoprotein | 2.26 | 2.39 | 2.51 | 3.07 | 0.01 |
| SAUSA300_1741 | putative lipoprotein | #N/A | #N/A | #N/A | #N/A | 1.63 |
| SAUSA300_1742 | hypothetical protein | -0.21 | -0.18 | -1.04 | -0.52 | 2.41 |
| SAUSA300_1743 | hypothetical protein | 2.64 | 4.06 | 4.81 | 4.87 | 0.47 |
| SAUSA300_1744 | hypothetical protein | 2.77 | 2.77 | 2.91 | 3.63 | 0.53 |
| SAUSA300_1745 | hypothetical protein | 2.78 | 2.28 | 1.68 | 1.64 | 1.81 |
| SAUSA300_1746 | hypothetical protein | 0.30 | 0.84 | 0.95 | 0.84 | 1.86 |
| SAUSA300_1747 | hypothetical protein | 0.71 | 0.47 | 1.40 | 1.22 | 1.96 |
| SAUSA300_1749 | hypothetical protein | #N/A | #N/A | #N/A | #N/A | -0.09 |
| SAUSA300_1750 | hypothetical protein | -0.25 | 0.06 | 0.09 | -0.19 | 0.93 |
| SAUSA300_1751 | type I restriction-modification enzyme, S subunit | #N/A | #N/A | #N/A | #N/A | -11.03 |
| SAUSA300_1752 | type I restriction-modification system, M subunit | #N/A | #N/A | #N/A | #N/A | -1.37 |
| SAUSA300_1754 | serine protease SplE | -0.67 | -0.32 | -0.68 | -1.37 | -5.58 |
| SAUSA300_1756 | serine protease SplC | #N/A | #N/A | #N/A | #N/A | -6.32 |
| SAUSA300_1757 | serine protease SplB | #N/A | #N/A | #N/A | #N/A | -5.96 |
| SAUSA300_1758 | serine protease SplA | #N/A | #N/A | #N/A | #N/A | -7.12 |
| SAUSA300_1759 | hypothetical protein | #N/A | #N/A | #N/A | #N/A | -6.54 |
| SAUSA300_1760 | lantibiotic epidermin immunity protein F | #N/A | #N/A | #N/A | #N/A | 2.61 |
| SAUSA300_1761 | lantibiotic epidermin immunity protein F | -1.23 | -0.66 | -0.41 | -0.43 | 1.40 |
| SAUSA300_1762 | lantibiotic epidermin immunity protein F | -0.26 | -0.76 | -0.28 | -0.31 | 1.76 |
| SAUSA300_1763 | lantibiotic epidermin leader peptide processing serine protease EpiP | -0.37 | -0.83 | -0.98 | -0.12 | 1.21 |
| SAUSA300_1764 | lantibiotic epidermin biosynthesis protein EpiD | -0.03 | 0.16 | -0.22 | 0.30 | 0.04 |
| SAUSA300_1765 | lantibiotic epidermin biosynthesis protein EpiC | #N/A | #N/A | #N/A | #N/A | 7.59 |
| SAUSA300_1766 | lantibiotic epidermin biosynthesis protein EpiB | #N/A | #N/A | #N/A | #N/A | 1.80 |
| SAUSA300_1767 | lantibiotic epidermin biosynthesis protein EpiA | -0.11 | -0.37 | -0.45 | -0.27 | 1.65 |
| SAUSA300_1768 | leukotoxin LukD | -0.11 | -0.06 | 0.63 | -0.01 | -1.71 |
| SAUSA300_1769 | leukotoxin LukE | #N/A | #N/A | #N/A | #N/A | 1.19 |
| SAUSA300_1770 | hypothetical protein | #N/A | #N/A | #N/A | #N/A | 2.40 |

*Negative values (green shading) represent genes higher expressed in USA400 isolate 649 compared to USA300 isolate 923. Positive values (red shading) represent genes higher expressed in USA300 isolate 923 compared to USA400 Isolate 649. #N/A indicate no expression ratio was obtained between isolates. Values are log2 ratios of differential gene expression.
