# Supplementary material for: Genomic and transcriptomic differences in community acquired methicillin resistant Staphylococcus aureus USA300 and USA400 strains
Source: BMC Genomics. 2014 Dec 19;15:1145. doi: 10.1186/1471-2164-15-1145 (PMC4630920; doi:10.1186/1471-2164-15-1145)
Supplement: Supplementary file 8 [file 12864_2014_7077_MOESM8_ESM.docx]

Table 2. Genes encoded in prophage ΦSa2usa

USA300	COMMON NAME	SYMBOL	30	60	90	120	in vivo
SAUSA300_1381	Panton-Valentine leukocidin, LukF-PV	lukF-PV	-1.015	-1.008	-0.547	-0.960	-1.411
SAUSA300_1382	Panton-Valentine leukocidin, LukS-PV	lukS-PV	0.328	-0.336	0.076	0.024	-2.711
SAUSA300_1383	phiSLT ORF484-like protein, lysin		-3.780	-4.184	-4.536	-4.829	-1.201
SAUSA300_1384	phiSLT ORF100b-like protein, holin		-2.240	-2.372	-2.266	-1.924	1.659
SAUSA300_1385	phiSLT ORF 99-like protein		-2.369	-2.209	-2.143	-2.548	-0.466
SAUSA300_1387	phiSLT ORF129-like protein		-2.466	-2.200	-2.376	-1.790	-1.321
SAUSA300_1389	phiSLT ORF636-like protein		N/A	N/A	N/A	N/A	-0.546
SAUSA300_1390	phiSLT ORF96-like protein		N/A	N/A	N/A	N/A	-0.156
SAUSA300_1391	phiSLT ORF527-like protein		-0.499	-0.612	-0.142	-0.594	1.134
SAUSA300_1392	phiSLT ORF191-like protein		-0.190	-0.800	-0.341	-0.817	1.424
SAUSA300_1393	phiSLT ORF2067-like protein, phage tail tape measure protein		N/A	N/A	N/A	N/A	3.239
SAUSA300_1394	hypothetical protein		-2.281	-0.902	-0.856	-0.678	0.419
SAUSA300_1395	phiSLT ORF116b-like protein		N/A	N/A	N/A	N/A	-0.646
SAUSA300_1396	phiSLT ORF151-like protein, major tail protein		-2.847	-2.906	-2.870	-2.440	-0.471
SAUSA300_1397	phiSLT ORF213-like protein, major tail protein		-4.036	-4.020	-3.868	-4.407	-0.196
SAUSA300_1398	phiSLT ORF123-like protein		-1.341	-2.494	-0.844	-1.375	4.124
SAUSA300_1399	phiSLT ORF110-like protein		-1.669	-1.221	-1.055	-2.650	-0.126
SAUSA300_1400	phiSLT ORF92-like protein, uncharacterized phage protein (DNA packaging)		-3.952	-4.259	-3.880	-3.927	-0.831
SAUSA300_1401	phiSLT ORF387-like protein, putative phage capsid protein		N/A	N/A	N/A	N/A	0.164
SAUSA300_1402	phiSLT ORF257-like protein, putative prophage protease		-3.403	-4.230	-4.539	-4.657	0.149
SAUSA300_1403	phiSLT ORF412-like protein, portal protein		-3.300	-2.855	-3.327	-2.734	-0.361
SAUSA300_1404	phiSLT ORF 563-like protein, terminase, large subunit		1.039	1.555	1.522	0.858	0.089
SAUSA300_1405	phiSLT ORF 101-like protein, terminase, small subunit		N/A	N/A	N/A	N/A	-0.031
SAUSA300_1406	phiSLT ORF 104b-like protein		-2.660	-2.128	-1.914	-1.841	N/A
SAUSA300_1407	phi SLT ORF 145-like protein, phage transcriptional regulator		-1.003	-0.664	-0.825	-0.856	-14.081
SAUSA300_1408	phage helicase		N/A	N/A	N/A	N/A	4.254
SAUSA300_1409	hypothetical protein		N/A	N/A	N/A	N/A	2.449
SAUSA300_1410	virulence-associated protein E		-1.030	-1.705	-1.553	-2.274	0.644
SAUSA300_1411	phiSLT ORF66-like protein		-0.906	-0.922	0.076	-0.046	1.464
SAUSA300_1412	phiSLT ORF 50-like protein		-0.971	-0.813	-0.127	-0.018	-0.236
SAUSA300_1415	phiSLT ORF 77-like protein		0.806	0.815	1.218	0.783	24.499
SAUSA300_1418	phiSLT ORF 82-like protein		-1.058	-1.241	-1.243	-1.531	4.844
SAUSA300_1419	phiSLT ORF80-like protein		-0.150	0.730	0.560	0.575	-1.051
SAUSA300_1421	phiSLT ORF122-like protein, DNA polymerase		N/A	N/A	N/A	N/A	-1.091
SAUSA300_1422	phiSLT ORF65-like protein		N/A	N/A	N/A	N/A	1.009
SAUSA300_1423	phage related DNA polymerase family A	polA	N/A	N/A	N/A	N/A	-2.386
SAUSA300_1424	hypothetical protein		-2.120	-1.618	-2.001	-1.061	1.124
SAUSA300_1425	hypothetical protein		N/A	N/A	N/A	N/A	-1.096
SAUSA300_1426	hypothetical protein		-1.411	-1.370	-1.402	-1.359	1.309
SAUSA300_1428	hypothetical protein		N/A	N/A	N/A	N/A	1.194
SAUSA300_1429	phiSLT ORF53-like protein		-1.590	-1.363	-2.148	-1.704	2.419
SAUSA300_1430	phiSLT ORF 87-like protein, putative DNA-binding protein		N/A	N/A	N/A	N/A	1.504
SAUSA300_1431	phiSLT ORF71-like protein		0.395	0.879	0.980	0.852	0.619
SAUSA300_1433	putative phage regulatory protein		N/A	N/A	N/A	N/A	0.779
SAUSA300_1434	phiSLT ORF104a-like protein, repressor		4.006	5.384	4.664	5.140	0.344
SAUSA300_1435	phiSLT ORF153-like protein		3.980	4.470	4.569	5.170	8.019
SAUSA300_1436	phiSLT ORF144-like protein, putative lipoprotein		N/A	N/A	N/A	N/A	0.804
SAUSA300_1437	phiSLT ORF204-like protein		3.411	4.788	4.173	4.064	0.854
SAUSA300_1438	phiSLT ORF401-like protein, integrase		1.081	1.147	1.211	0.833	1.684

*Negative values (green shading) represent genes higher expressed in USA400 isolate 649 compared to USA300 isolate 923. Positive values (red shading) represent genes higher expressed in USA300 isolate 923 compared to USA400 Isolate 649. #N/A indicate no expression ratio was obtained between isolates. Values are log2 ratios of differential gene expression.
