# Supplementary material for: Genomic and transcriptomic differences in community acquired methicillin resistant Staphylococcus aureus USA300 and USA400 strains
Source: BMC Genomics. 2014 Dec 19;15:1145. doi: 10.1186/1471-2164-15-1145 (PMC4630920; doi:10.1186/1471-2164-15-1145)
Supplement: Supplementary file 9 [file 12864_2014_7077_MOESM9_ESM.doc]

Table 3. Genes encoded in prophage ΦSa3usa

USA300	COMMON_NAME	SYMBOL	30	60	90	120	in vivo
SAUSA300_1919	hypothetical protein		0.249	-0.145	-1.170	-1.737	-3.836
SAUSA300_1920	chemotaxis-inhibiting protein CHIPS	chs	#N/A	#N/A	#N/A	#N/A	13.114
SAUSA300_1921	truncated amidase		-1.274	-0.867	-0.858	-1.736	5.314
SAUSA300_1922	staphylokinase precursor	sak	-0.464	0.007	-0.293	0.549	2.644
SAUSA300_1923	autolysin		-0.726	-0.366	-0.631	-0.789	4.879
SAUSA300_1925	phiPVL ORF17-like protein		#N/A	#N/A	#N/A	#N/A	0.389
SAUSA300_1926	phi77 ORF044-like protein		3.596	4.314	4.883	4.819	8.594
SAUSA300_1927	phi77 ORF109-like protein		1.965	1.185	1.204	0.984	2.809
SAUSA300_1928	phi77 ORF002-like protein, phage minor structural protein		2.748	3.445	4.294	2.407	-1.076
SAUSA300_1929	phi77 ORF004-like protein, putative phage tail component		2.398	2.731	2.697	3.494	3.749
SAUSA300_1930	phi77 ORF001-like protein, phage tail tape measure protein		2.794	4.217	3.975	3.213	2.634
SAUSA300_1931	phi77 ORF100-like protein		3.474	3.449	3.521	3.180	-1.461
SAUSA300_1932	hypothetical protein		3.552	4.854	5.313	5.553	#VALUE!
SAUSA300_1933	hypothetical protein		#N/A	#N/A	#N/A	#N/A	2.139
SAUSA300_1934	phi77 ORF020-like protein, phage major tail protein		3.749	4.395	4.424	3.574	5.759
SAUSA300_1935	phi77 ORF029-like protein		2.948	5.024	4.454	3.439	8.489
SAUSA300_1936	hypothetical protein		3.904	5.142	5.273	4.063	5.534
SAUSA300_1937	phi77 ORF045-like protein		3.761	2.508	1.545	2.744	#VALUE!
SAUSA300_1938	phi77 ORF006-like protein, putative capsid protein		4.225	4.663	3.312	4.604	2.189
SAUSA300_1939	phi77 ORF015-like protein, putative protease		2.443	2.844	2.192	1.672	4.974
SAUSA300_1940	phage portal protein		3.151	3.834	3.290	2.677	5.614
SAUSA300_1941	phi77 ORF003-like protein, phage terminase, large subunit		#N/A	#N/A	#N/A	#N/A	2.489
SAUSA300_1942	hypothetical protein		1.883	2.675	2.614	3.307	2.369
SAUSA300_1943	phi77 ORF040-like protein		-0.227	1.395	2.167	0.958	9.929
SAUSA300_1944	phi77 ORF026-like protein, putative phage transcriptional activator		#N/A	#N/A	#N/A	#N/A	3.224
SAUSA300_1945	phi77 ORF071-like protein		-0.743	0.049	0.165	0.488	-2.846
SAUSA300_1950	hypothetical protein		2.428	3.231	3.331	2.419	3.959
SAUSA300_1951	phiPVL ORF052-like protein		1.275	1.533	1.320	0.599	2.744
SAUSA300_1952	phiPV083 ORF027-like protein		2.206	3.731	3.537	3.475	4.034
SAUSA300_1954	phiPVL ORF050-like protein		1.897	1.032	1.041	-0.351	-0.126
SAUSA300_1956	hypothetical protein		#N/A	#N/A	#N/A	#N/A	3.914
SAUSA300_1957	phiPVL ORF046-like protein		#N/A	#N/A	#N/A	#N/A	1.764
SAUSA300_1958	single-strand binding protein		-1.386	-1.025	-1.050	-0.888	1.779
SAUSA300_1959	phiPVL ORF044-like protein		#N/A	#N/A	#N/A	#N/A	6.624
SAUSA300_1960	putative phage-related DNA recombination protein		#N/A	#N/A	#N/A	#N/A	3.389
SAUSA300_1961	phiPVL ORF41-like protein		-0.126	0.207	0.109	-0.244	3.479
SAUSA300_1963	hypothetical protein		#N/A	#N/A	#N/A	#N/A	3.459
SAUSA300_1964	hypothetical protein		-1.742	-1.971	-1.711	-2.542	6.534
SAUSA300_1967	hypothetical protein		#N/A	#N/A	#N/A	#N/A	10.129
SAUSA300_1968	putative phage transcriptional regulator		#N/A	#N/A	#N/A	#N/A	8.119
SAUSA300_1969	phi77 ORF011-like protein, phage transcriptional repressor		3.260	4.127	4.218	4.329	10.979
SAUSA300_1970	putative exonuclease		#N/A	#N/A	#N/A	#N/A	4.229
SAUSA300_1971	phi77 ORF017-like protein		3.797	3.227	2.679	3.159	#N/A
SAUSA300_1972	integrase	int	#N/A	#N/A	#N/A	#N/A	4.379

*Negative values (green shading) represent genes higher expressed in USA400 isolate 649 compared to USA300 isolate 923. Positive values (red shading) represent genes higher expressed in USA300 isolate 923 compared to USA400 Isolate 649. #N/A indicate no expression ratio was obtained between isolates. Values are log2 ratios of differential gene expression.
